# Evaluation of a disability-inclusive ultra-poor graduation programme in Bangladesh: study protocol for a cluster-randomised controlled trial

**DOI:** 10.1186/s13063-026-09800-6

**Published:** 2026-05-23

**Authors:** Mark T. Carew, Semab Rahman, Lena Morgon Banks, Mohima Gomes, Md. Kamruzzaman, Narayan Das, Hannah Kuper

**Affiliations:** 1 International Centre for Evidence in Disability, London School of Hygiene & Tropical Medicine, London, UK; 2 BRAC Institute of Governance and Development, BRAC University, Dhaka, Bangladesh

**Keywords:** Disability, Financial support, Income generation, Livelihoods, Social protection, Randomised controlled trial

## Abstract

**Background:**

There remains a lack of evidence on which livelihoods interventions are effective for persons with disabilities, particularly given the fact that the impact of interventions varies according to contextual factors. The disability-inclusive ultra-poor graduation (DIUPG) programme is an adaptation of the ultra-poor graduation programme (UPG), designed to tackle extreme poverty among persons with disabilities. The DIUPG programme has been previously evaluated in Uganda and found to have successfully improved household consumption, amidst the challenges of COVID-19 and funding cuts. The current evaluation of DIUPG programme therefore creates the opportunity to generate comparative data on the programme in a different context (Bangladesh), which will aid understanding of the transferability and robustness of the intervention.

**Methods:**

The DIUPG provides standard UPG support such as asset transfers, combined with disability-specific elements such as rehabilitation and psychosocial therapy. The evaluation will adopt a cluster-randomised superiority trial design with two parallel groups, with clusters comprising branch office catchment areas of the intervention implementer. The units of participation in DIUPG are households living in these geographical areas. Household eligibility is determined through household screening utilising standardised measures to identify ultra-poor households that have members with disabilities. Clusters are randomly selected prior to DIUPG implementation, resulting in 48 intervention and 24 control clusters. The primary outcomes of the evaluation are per capita household income, income and employment of persons with disabilities, per capita food consumption and expenditures, and per capita non-food expenditures. Prior to intervention implementation, baseline data is collected in the treatment and control arms (May–June 2023). After DIUPG conclusion (20 months), endline data are collected (October–November 2025) in both arms.

**Discussion:**

There is a general paucity of evidence regarding what interventions work to create better livelihoods for persons with disabilities and their families. Evaluating the DIUPG programme in Bangladesh will generate crucial evidence demonstrating whether similar benefits of the programme as observed in Uganda accrue in a distinct context.

**Trial registration:**

ISRCTN registry ISRCTN10602983. Retrospectively registered on 05/05/2025.

**Supplementary Information:**

The online version contains supplementary material available at 10.1186/s13063-026-09800-6.

## Administrative information

Note: the numbers in curly brackets in this protocol refer to SPIRIT checklist item numbers. The order of the items has been modified to group similar items (see http://www.equator-network.org/reporting-guidelines/spirit-2013-statement-defining-standard-protocol-items-for-clinical-trials/).

**Table:** 

Title {1}	Evaluation of a disability-inclusive ultra-poor graduation programme in Bangladesh: study protocol for a cluster-randomised controlled trial
Trial registration {2a and 2b}	ISRCTN registry ISRCTN10602983 Retrospectively registered on 05/05/2025
Protocol version {3}	Version 2 (05/03/2026)
Funding {4}	The study is funded by the United Kingdom Foreign, Commonwealth and Development Office under the Programme for Evidence to Inform Disability Action (PENDA) (IATI Identifier: GB-EDU-133903-PENDA), with additional funding support for the baseline data collection provided by BRAC. The intervention is designed and implemented by BRAC under a separate project, funded by the UK and Australian governments.
Author details {5a}	Mark T. Carew [1]* & Semab Rahman [2]* (joint first), Lena Morgon Banks [1], Mohima Gomes [2], Md. Kamruzzaman [2], Narayan Das [2]* & Hannah Kuper* [1] (joint last) [1] International Centre for Evidence in Disability, London School of Hygiene & Tropical Medicine, London, United Kingdom; [2] BRAC Institute of Governance and Development, BRAC University, Dhaka, Bangladesh;
Name and contact information for the trial sponsor {5b}	Not applicable. This is an observational evaluation study that aims to evaluate to what extent the intervention has achieved its goals. The researchers are not involved in the design, allocation or implementation of the intervention—this is being led by BRAC.
Role of sponsor {5c}	Neither FCDO nor BRAC are not involved in any decision making with regard to the study design, implementation, analysis or publications.

## Introduction

### Background and rationale {6a}

The goal of eradicating poverty set out by the Sustainable Development Goal (SDGs) necessitates reaching the very poorest families and shifting them to sustainable income-generating activities that enable them to escape the poverty trap. However, despite initial enthusiasm about the prospect of eliminating poverty by 2030, there is growing evidence that the international community will fall short of this target [8]. This is sobering news for the world’s 1.3 billion persons with disabilities [12] in light of mounting evidence showing that they are likely to experience greater levels of poverty compared to peers without disabilities [3, 4, 10]. Effective poverty alleviation for persons with disabilities is complicated by a range of challenges to their inclusion that operate both within poverty reduction programmes and the contexts that they take place in. These challenges include widespread disability stigma and discrimination, environmental inaccessibility and the failure of these programmes to ensure disability-related adaptations or effectively mitigate disability-related extra costs [3, 4, 7].

One example of a poverty alleviation programme that has meaningfully included persons with disabilities is the disability-inclusive ultra-poor graduation (DIUPG) programme, implemented by the non-governmental organisation BRAC. The programme is an adaptation of the ultra-poor graduation programme (UPG), which targets extreme poverty [7]. There is evidence that the UPG approach is effective at alleviating poverty. Specifically, randomised controlled trials of the UPG programme that were conducted in six countries (Ethiopia, Ghana, Honduras, India, Pakistan, and Peru) showed it improved the lives of beneficiaries across several key poverty-related indices, including by increasing consumption, food security and asset ownership [1]. Importantly, as this example shows, poverty is multidimensional, encompassing several economic domains, which the UPG programme is designed to impact. The key feature of the UPG programme is transfer of productive assets, aimed to help families improve their economic situation. The UPG programme combines asset provision with broader support, including regular cash or food consumption assistance, skills training and opportunities for savings [1]. UPGs have often included persons with disabilities (4–5% of recipients; unpublished data), although programme implementers have recognised that the mainstream model required adaptations to ensure that persons with disabilities can enrol and benefit from the programme. The disability-inclusive version of the programme introduces additional disability-specific programme components that aim to tackle the unique barriers that obstruct persons with disabilities and their families from escaping the trap of poverty, such as accessible trainings and access to rehabilitation, assistive products and other supports.

To date, BRAC has adapted the UPG programme to be disability-inclusive in Uganda. The disability-inclusive ultra-poor graduation programme (DIUPG) in Uganda ran for 18 months from December 2020 to June 2022 and has already undergone an impact and process evaluation. Findings were encouraging, suggesting that the DIUPG Uganda programme successfully improved household assets, consumption and incomes among households with members with disabilities. Currently, BRAC has funding to roll out the disability-inclusive UPG programme in Bangladesh through financial support provided by a strategic partnership agreement between BRAC and the Australian and UK governments. This is a crucial opportunity to evaluate whether similar benefits of the disability-inclusive ultra-poor graduation programme in Uganda are observed in Bangladesh. This is important because there remains a lack of evidence on effective livelihoods interventions for persons with disabilities [6]. Moreover, the effectiveness of interventions varies according to contextual factors, with evidence from the DIUPG Uganda evaluation suggesting the programme was detrimentally affected by COVID-19 and associated funding cuts, for instance [9]. Another evaluation of DIUPG therefore creates the opportunity to generate comparative data on the programme in a different context (Bangladesh), which can be used to equip policymakers and programmers with a greater understanding of the transferability and robustness of the intervention.

Bangladesh is a highly suitable context in which to evaluate the DIUPG intervention. Bangladesh is a lower-middle-income country that has experienced sustained economic growth and reduction in extreme poverty since 2010 [11]. However, since 2016 poverty reduction has slowed, with consumption gains disproportionately benefitting middle- and higher-income groups, and limited improvement among the poorest households [11]. Approximately 7% of Bangladesh’s population live with some form of disability [2]. Given that disability and poverty are linked [3, 4], many of these households experience extreme poverty.

The London School of Hygiene and Tropical Medicine (LSHTM) has funding from the United Kingdom’s Foreign Commonwealth and Development Office (FCDO) to conduct an impact evaluation and process evaluation of DIUPG Bangladesh through the Programme for Evidence to Inform Disability Action (PENDA) project. PENDA will evaluate the DIUPG Bangladesh programme, in partnership with BRAC Institute of Governance and Development (BIGD). BIGD is based at BRAC University, which operates independently of BRAC. As PENDA funding for the evaluation was limited, some funds were also given by BRAC for the baseline data collection.

### Objectives {7}

The impact evaluation will assess the effect of the DIUPG Bangladesh programme on the livelihoods and well-being of persons with disabilities and their families. The primary objective is to estimate the effect on poverty reduction and economic well-being, specifically through assessing changes in income, employment, and household consumption. The secondary objectives assess the impact of the programme on key dimensions of quality of life, including social inclusion, psychological well-being, financial access, and health-seeking behaviour.

### Trial design {8}

The evaluation will adopt a cluster-randomised superiority trial design with two parallel groups. Clusters are defined as BRAC branch office catchment areas. Within clusters, households act as the unit of participation. Cluster-randomisation is appropriate for the DIUPG Bangladesh intervention because some components of the project are delivered at the community level. The intervention has identified eligible households from 96 branch office catchment areas from 14 districts. BRAC further selected branch office catchment areas from two other districts but these two districts are not covered in the evaluation as the selection in those branch offices was delayed due to administrative reasons. All selected branch office catchment areas are located in rural areas of Bangladesh.

A baseline survey at household level was undertaken (June, 2023) after random allocation of BRAC branch office catchment areas into intervention and control arms, but prior to implementation of the intervention. The DIUPG Bangladesh programme is then implemented for 20 months from October 2023 to May 2025. An endline survey at household level will then be undertaken (October–November 2025).

The trial includes an integrated process evaluation which will examine implementation of the DIUPG Bangladesh programme, including the mechanisms by which the intervention operated and the influence of context on programme delivery and outcomes.

## Methods: participants, interventions and outcomes

### Study setting {9}

The DIUPG Bangladesh programme will be implemented within 14 districts of Bangladesh (Barguna, Gaibandha, Gopalgonj, Hobigonj, Jashore, Jhalokhati, Joypurhat, Kishoreganj, Kurigram, Lalmonirhat, Mymensingh, Pirojpur, Rangpur, Sirajgonj).

### Eligibility criteria {10}

For the purposes of the DIUPG Bangladesh intervention, clusters are defined as BRAC branch office catchment areas. These catchment areas correspond to geographical areas of typically a 5–6-km^2^ radius from BRAC branch offices. BRAC identified eligible ultra-poor households for the DIUPG Bangladesh intervention by screening households within the 96 branch office catchment areas in the 14 target districts. Specifically, eligible ultra-poor households were those that met the following conditions:

1. Monthly per capita household income ≤ BDT 2250 (approximately 21.83 USD at the nominal 2023 exchange rate)
2. Household has at least one member aged 18–62 years
3. Household has at least one person with disability who is aged 1–62 years
4. Household is excluded if any of the household members have an institutional loan (with exceptions at the discretion of BRAC).

Initial selection of eligible households is conducted via a two-step process. First, the programme adapts UPG’s participatory rural appraisal (PRA) method to divide the village population into 5–6 economic groups where the three poorest groups are shortlisted for selection in DIUPG. Next, an assessment of their economic vulnerability through a household questionnaire gathers data on household demographics, income, savings, loans, asset ownership, and land ownership. At the same time, the Washington Group Short Set (WGSS) of questions is used within this questionnaire to identify persons with disabilities. The WG SS is a set of 6 standardised questions designed to identify persons with disabilities by assessing functional difficulties in areas such as vision, hearing, mobility, cognition, and communication. All sets of measures help ensure households meet the DIUPG selection criteria and also undergo further verification by BRAC staff. Specifically, to verify disability status, trained rehabilitation and psychosocial professionals conduct a final assessment to evaluate the physical and psychological functioning of individuals with disabilities, ensuring accurate final identification.

All households selected for the DIUPG Bangladesh programme contain a person with a disability. The unit of participation in the programme is the household; however, a single individual within each household is the ‘project participant’ who is the primary recipient of the assets and rehabilitation and psychosocial support delivered by the DIUPG Bangladesh programme. All project participants in DIUPG are persons with disabilities (i.e. the pre-identified index individual within each enrolled household who is the target beneficiary of the intervention components). In the majority of cases, project participants take direct responsibility for management of the provided asset and participate in programme activities, such as enterprise development training or savings group meetings. However, where they are individuals with severe disabilities or children, management of the asset and participation in some project activities (e.g. Village Social Solidarity Committee meetings) is undertaken by their caregiver or another family member. If a household contains multiple individuals with disabilities, only one is registered as the primary participant. Other persons with disabilities in the household may receive support (e.g. rehabilitation and psychosocial training) where relevant, but they are not formal programme participants and do not receive livelihood assets.

### Who will take informed consent? {26a}

Informed consent is obtained at each round of data collection. The informed consent process is conducted by the BIGD interviewer, trained in the informed consent and wider research process. Before taking informed consent, the interviewer provides hard copies and reads aloud the participant information sheet and consent forms to the participants. Consent and data collection procedures are adapted to support the participation of people with different impairments. In cases where individuals with disabilities are unable to provide informed consent (e.g. persons with intellectual disabilities), their caregivers provide consent on their behalf. Written informed consent is then obtained by means of participant-dated signature (or thumbprint for those who cannot read) and dated signature of the person who obtained the consent.

### Additional consent provisions for collection and use of participant data and biological specimens {26b}

Not applicable. There are no additional provisions as participant data is collected for use in this study only.

## Interventions

### Explanation for the choice of comparators {6b}

Participants in the control arm do not receive any intervention but are expected to receive the DIUPG intervention after the trial. BRAC identified eligible households for both the regular UPG programme and disability-inclusive ultra-poor programme in both the treatment and control branch office catchment areas. In the treatment arm, DIUPG and UPG run concurrently, with households who are eligible for DIUPG receiving the DIUPG programme and households that are eligible for the regular UPG programme receiving UPG support. In the control arm, no households within branch office catchment areas receive either DIUPG or UPG. Hence, households eligible for the DIUPG programme are unlikely to be indirectly affected by the regular UPG programme in the control branches.

### Intervention description {11a}

The DIUPG Bangladesh programme is implemented similarly to how the DIUPG programme was implemented in Uganda. Specifically, DIUPG Bangladesh takes a twin-track approach. First, it aims to ensure that the mainstream components of the ultra-poor graduation approach reach persons with disabilities. Second, it adds targeted activities designed to meet the unique needs of persons with disabilities. The four main ultra-poor graduation programme components of DIUPG Bangladesh are:

1. Livelihood: Participants receive assets, technical training on enterprise and skill development, linkage to local markets and other individual-level support (e.g. household visits). Assets are provided based on a prior assessment of local market opportunities and the skills and capabilities of the participants, conducted at the eligibility verification stage. Participants are further divided into two groups based on their economic vulnerability. The most economically vulnerable group is given the full asset without any repayment obligation. The other group receives the asset but is required to repay 50% of its value in 36 interest-free instalments. The livelihood component is expected to lead to improved enterprise management skills, increased productive assets, and increased access to local markets via products and jobs.
2. Social protection: Participants receive support in accessing relevant government services, including social protection, healthcare programmes, and other forms of assistance. The social protection component aims to increase awareness and improve access to these services. It also helps beneficiaries overcome barriers to both disability-specific and general social entitlements,
3. Financial inclusion: Participants receive financial literacy training to support savings behaviour and BRAC creates platforms for savings. Each member can maintain a savings account where they can deposit funds based on their financial capacity and anticipated future needs. There is no fixed amount required for saving. The programme will raise awareness about the importance of saving and encourage members to save regularly. Members will also earn yearly interest on their savings, as per the programme regulations. Financial inclusion is expected to result in improved financial management skills and greater access to financial services.
4. Social empowerment: Intervention activities include home coaching for individuals, awareness raising on general issues (e.g. health and hygiene) and the formation of Village Social Solidarity Committees (VSSCs). These are local governance structures with government/community leadership representation, comprised of people with good reputations, who are known for their community service. Although it is a mainstream component of UPG, DIUPG participants also attend them and the VSSC provides additional support to the DIUPG households as needed. Specifically, through regular discussions, the VSSC identifies the needs of DIUPG participants and works collaboratively to connect them with appropriate government and non-government programmes. As such, the committee helps establish linkages with relevant institutions and stakeholders to ensure participants receive the assistance they need. Social empowerment is expected to result in better social involvement and participation within households and communities, improving participant confidence and aspirations.

In terms of disability-specific activities, the DIUPG intervention provides (1) access to rehabilitation and psychosocial support and may include referrals to health professionals. Specifically, rehabilitation support includes physiotherapy, assistive device provision and adaptations to the individual’s home and environment to help them with daily tasks. Psychosocial support includes psychosocial therapy inclusive life-skills training and other forms of coaching and emotional support. All these help to address individual barriers related to disability among DIUPG participants. It also provides (2) training to BRAC staff facilitated by local Organisations of Persons with Disabilities (OPDs) designed to improve disability inclusion at the organisational level. This is done through a disability inclusion self-assessment of BRAC, development of a disability inclusion action plan and monitoring of implementation. Finally, (3) the DIUPG Bangladesh programme conducts sensitisation of village leaders and other key stakeholders on disability inclusion facilitated by local OPDs. The DIUPG programme also ensures that local OPD leaders are represented in VSSC membership. The purpose of these advocacy activities is to shift community norms and behaviour around disability inclusion.

The presence of these advocacy activities, and the VSSCs, means that while the DIUPG Bangladesh programme is predominantly a household-level intervention, it also has important village-level components.

**Fig. 1:**
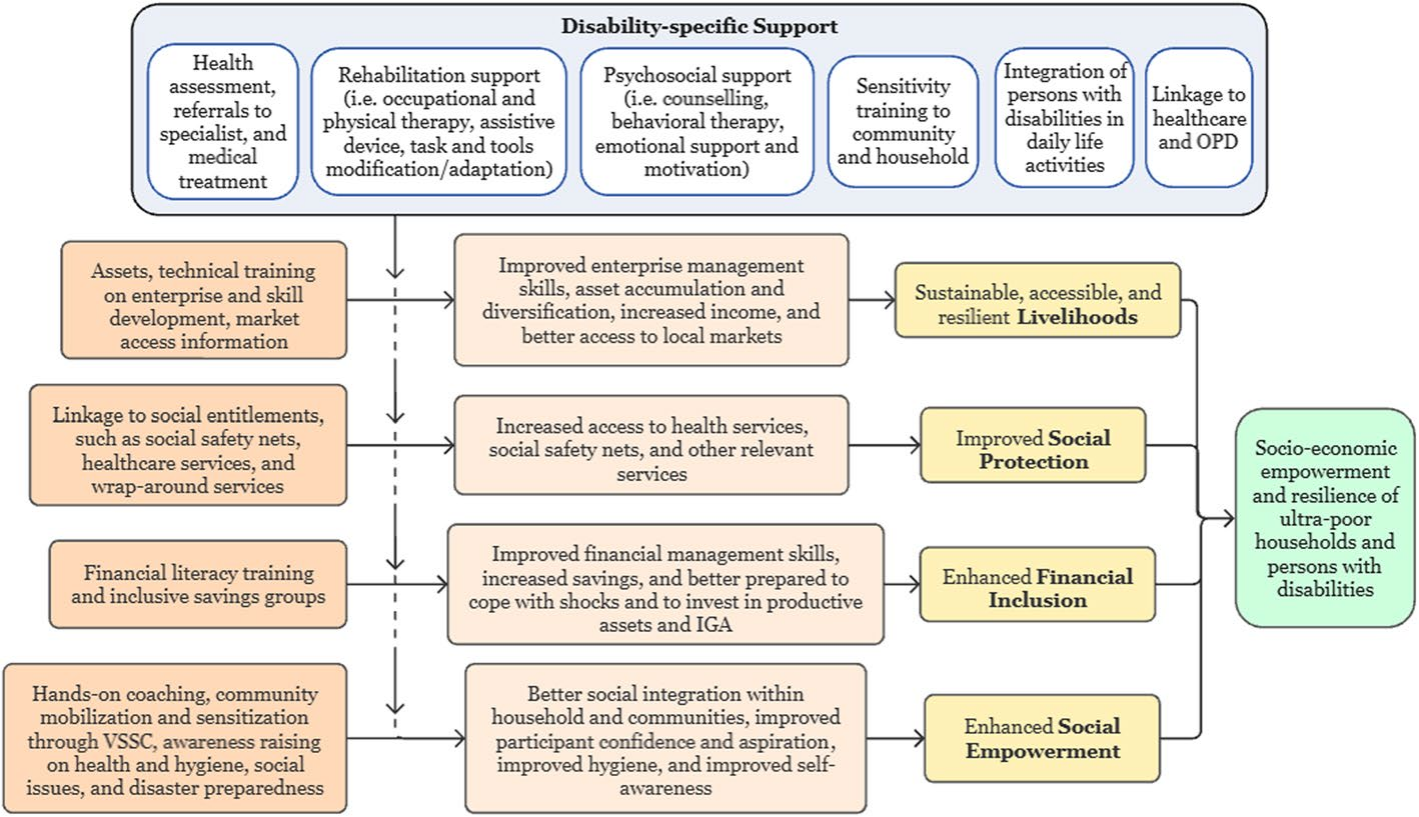
DIUPG Bangladesh programme theory of change

The DIUPG Bangladesh programme theory of change (ToC) is shown in Fig. 1.

Sustainability within DIUPG refers to the extent to which economic gains persist beyond the duration of intervention activities. The programme assumes that mainstream UPG components such as productive asset provision will enable households to obtain stable gains and avoid re-entry into extreme poverty once support is withdrawn. Moreover, as the programme is being conducted in a disability context, its sustainability depends not only on addressing economic barriers but also on whether disability-related barriers such as the inaccessibility of the environment are persistently mitigated. The function of disability-specific components is to sustainably address these barriers.

### Criteria for discontinuing or modifying allocated interventions {11b}

DIUPG Bangladesh programme participants and households can choose to stop their participation in the intervention or evaluation at any time without having to give a reason and without impacting the programmes or services that BRAC offers to them in the future. No modification of allocated interventions will be conducted.

### Strategies to improve adherence to interventions {11c}

BRAC monitors the implementation of interventions and the commitment to programme activities, ensuring they are carried out in a timely and appropriate manner. The approach is somewhat adaptive, as BRAC generates recommendations every 3 months on the implementation of interventions. The programme adopts those recommendations that are feasible and relevant, allowing adjustments to be made as needed in response to identified issues.

### Relevant concomitant care permitted or prohibited during the trial {11d}

There are no restrictions on concomitant care.

### Provisions for post-trial care {30}

There are no specific provisions for post-trial care.

### Outcomes {12}

As poverty is multidimensional, this evaluation therefore specifies five primary outcomes across key economic dimensions aligned with the DIUPG Theory of Change. The five primary outcomes focus on (1) per capita household income, (2) income of persons with disabilities, (3) employment of persons with disabilities, (4) per capita food consumption and expenditures, and (5) per capita non-food expenditures. Secondary outcomes include financial inclusion, assessed through savings and credit access for persons with disabilities and other household members; psychological and mental well-being; health-seeking behaviour; and social inclusion of persons with disabilities. Individual-level outcomes are collected for the household’s index participant with disability only (i.e. the designated DIUPG participant or their equivalent).

### Participant timeline {13}

Table 1 shows the participant timeline, and Table 2 shows DIUPG Bangladesh programme activities.

**Table 1:**
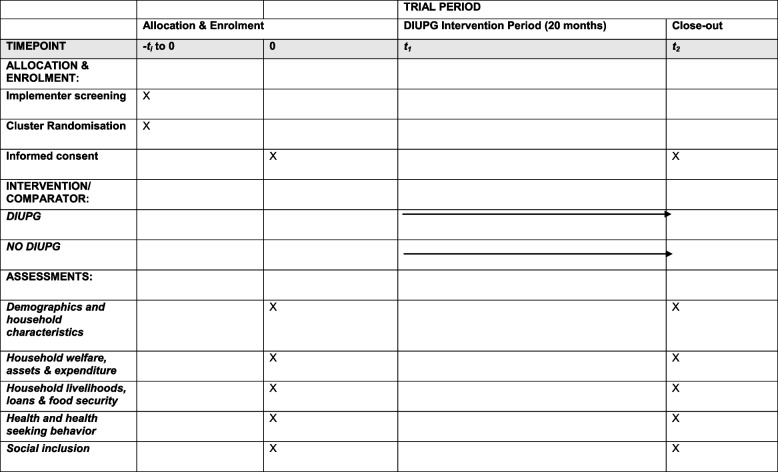
Participant timeline: schedule of enrolment, interventions, and assessments

**Table 2:** Outline of intervention activities over 20 months of the DIUPG Bangladesh programme

Month 1 to 5	Month 6–10	Month 11–15	Month 16–20
Enterprise and skill development training Asset transfer Health assessment, referrals and rehabilitation and psychosocial support to person with disability One-to-one follow-up and supervision of enterprise and health condition of person with disability Community awareness building Formation of the VSSC Linkage creation	Guidance on asset diversification One-to-one follow-up and supervision of enterprise and health condition of person with disability Community awareness building (i.e. early marriage, dowry, waterborne disease, health and hygiene practise) Ongoing rehabilitation and psychosocial support Savings and payable grant instalment collection Linkage with other services (government and nongovernment) VSSC meeting participation Referral activities	Enterprise and skill development training One-to-one follow-up and supervision of enterprise and health condition of person with disability Ongoing rehabilitation and psychosocial support VSSC meeting participation Referral activities	Formal weekly meetings Payable activities Savings activities One-to-one follow-up and supervision of enterprise and health condition of person with disability Ongoing rehabilitation and psychosocial support Introduction to the regular Microfinance Programme

### Sample size {14}

The sample size calculations are derived from the estimated effect of the intervention on the primary outcome of per-capita monthly household income among persons with disabilities. The total number of households participating in the DIUPG Bangladesh programme equates to roughly 35 households per cluster (i.e. BRAC branch office catchment area). The intraclass correlation coefficient (ICC) is calculated from baseline data collected for the evaluation of a disability-inclusive training programme in Bangladesh [5] provide detailed information on the evaluation and data collection process for this training programme. The total number of clusters in our study is 72; it is the number of households per cluster that we have the most capacity to vary. The minimum detectable effect (MDE) was specified as a difference of 0.20 standard deviations in per-capita income, expressed in SD units. This reflects the smallest effect size considered to be policy-relevant in this context. For comparison, a recent quasi-experimental study on the 2017 cohort of BRAC UPG programme estimated an effect on per capita income that was quite considerably larger (nearly 0.50 SD) (Arman MR, Bhattacharjee A, Das N, Matin I, Rahman A. Getting the loan-grant blend right: how effective is the segmentation approach for the ultra-poor graduation program?, unpublished). With an intra-cluster correlation of 0.055 and effect size of 0.20 SD, the sample size of 35 households per cluster gives us a power of 0.80. This produces a total sample size for this evaluation of 2520 households, equating to approximately 1680 people with disabilities in the intervention clusters and 840 in the control.

### Recruitment {15}

BRAC is responsible for the selection of households to take part in the programme. This process is based on household-level indicators, operating across two stages:

Stage 1: Initial screening of potentially eligible households. The programme divides the village population into 5–6 economic groups as previously described where the three poorest groups are shortlisted for selection in DIUPG.

Stage 2: Selection of eligible households. BRAC screens selected households in branch office catchment areas in the target districts. Eligible households are identified based on the eligibility criteria, as earlier described. Verification is completed by the project manager of the DIUPG Bangladesh programme. The project manager verifies that households meet the eligibility criteria, as earlier described.

The unit of participation in the programme is the household; however, a single individual within each household is the ‘project participant’ who is the main recipient of the training and assets. This person is expected to take responsibility for managing the enterprise and participate in other activities, such as training on enterprise management or savings group meetings. Project participants are selected before randomisation.

## Assignment of interventions: allocation

### Sequence generation {16a}

Randomisation for the evaluation of the DIUPG programme is stratified without blocking by district to ensure regional balance. BRAC initially identified 16 districts for intervention implementation. However, for randomisation, two districts were excluded due to delays in their selection process, leaving 14 districts for the evaluation. Specifically, BRAC identified 96 branch office catchment areas (i.e. clusters) across the 14 districts. From these, 72 branch out of 96 office catchment areas were randomly selected through stratified random sampling by district in Stata, due to limited evaluation funding.

### Concealment mechanism {16b}

No concealment mechanism, the random allocation of branches to the treatment and control groups is conducted using Stata.

### Implementation {16c}

The assignment of branch office catchment areas to the intervention and control groups was conducted prior to the baseline survey and programme rollout. This process, carried out by the research team, was designed to align with the programme’s feasibility and evaluation objectives. Within each district, the 72 branch office catchment areas were randomly assigned in a single step to either the treatment or control group, stratified by district and without block randomisation. We included 3 or 6 branch office catchment areas from each district to maintain a 2:1 ratio between the treatment and control groups. In districts where 3 branch office catchment areas were selected, one was randomly assigned to the control arm and the remaining two to the treatment arm. In districts with 6 branch office catchment areas, two were randomly assigned to the control arm and four to the treatment arm.

Randomisation was performed using Stata, ensuring that 24 branch office catchment areas (one-third of the selected branches) were allocated to the control arm, while 48 were assigned to the treatment arm. In the remaining 24 branches not involved in the evaluation, BRAC also implemented DIUPG.

Once randomisation was complete, the baseline survey was conducted, then programme implementation proceeded according to the pre-assigned treatment groups. No reassignment of branches occurred after randomisation. During baseline data collection, neither the enumerators nor the local field staff were aware of which branch offices were assigned to the treatment or control arm.

## Assignment of interventions: blinding

### Who will be blinded {17a}

After households have been allocated to receive the DIUPG Bangladesh programme there is no masking as households and project participants in clusters assigned to intervention or control are aware of their allocation given the nature of the intervention.

### Procedure for unblinding if needed {17b}

Not applicable as there is no post-allocation masking.

## Data collection and management

### Plans for assessment and collection of outcomes {18a}

Baseline data is collected (May–June 2023) after randomisation but before the DIUPG Bangladesh programme starts. After the DIUPG Bangladesh programme ends following 20 months of implementation, endline data is collected (October–November 2025). Data is collected for the baseline and endlines using questionnaires that have been pilot tested. Questionnaires are administered by experienced and trained data collectors, supervised by BIGD, to participants within selected households in both the control and intervention arms. One questionnaire is administered to the individual best able to answer questions about the household (usually the head of the household); a second is administered to the project participant (i.e. the person with disabilities, who in some cases is the same person as the household head). Table 3 displays the key domains for each questionnaire.

**Table 3:** Key questionnaire domains

Household questionnaire	Individual questionnaire (if not household head)
Demographics and household characteristics Household welfare and assets Household expenditure Household agriculture, livelihoods, and other income-generating activities Household loans and savings Food security	Self-related health (including assistive device use), health seeking behaviour and psychological wellbeing Social inclusion encompassing: stigma and discrimination, involvement in the community and household decision making; social networks, environmental accessibility)

### Plans to promote participant retention and complete follow-up {18b}

The evaluation will employ standard good practice strategies to promote retention such as collecting multiple contact phone numbers at baseline and making multiple attempts to recontact participants.

### Data management {19}

All data collection for the evaluation is being led by BIGD. Quantitative data, collected through the household surveys, is collected electronically using SurveyCTO where all data is password protected on devices and servers. Data are cleaned and anonymised by BIGD, then shared with LSHTM in the UK using a secure data transfer protocol, where it is securely stored on LSHTM servers.

### Confidentiality {27}

All study staff undergo ethics training and sign a confidentiality agreement. The research team ensures that all research data collected are anonymised using unique identification numbers.

### Plans for collection, laboratory evaluation and storage of biological specimens for genetic or molecular analysis in this trial/future use {33}

Not applicable, as no biological specimens collected.

## Statistical methods

### Statistical methods for primary and secondary outcomes {20a}

We will not use formal hypothesis tests of baseline differences to assess the validity of randomisation, as any observed differences arise by chance under random assignment and do not constitute evidence of imbalance. Baseline characteristics will instead be presented by treatment arm using descriptive statistics (means and standard deviations for continuous variables; counts and proportions for categorical variables). Standardised mean differences will be reported to summarise the magnitude of any baseline differences.

For transparency and descriptive purposes, we will report estimated differences in baseline characteristics between arms using the following regression equation:

1 $${y}_{ibd}=\alpha +\upbeta {T}_{bd}+{\tau}_{d}+{\varepsilon}_{ibd}$$

where ${y}_{ibd}$ represents the baseline characteristics (primary and secondary outcomes, and some household/individual characteristics such as household size, age, sex of household head, etc.) of the individual or household *i* from branch office *b* in district *d*. ${T}_{bd}$ is an indicator variable taking the value of 1 if individual or household i is assigned to the treatment group and 0 if assigned to the control group. ${\tau}_{d}$ are district fixed effects. Since the randomisation is stratified at the district level, we control district fixed effects. ${\varepsilon}_{ibd}$ is the error term. We will cluster standard error by branch office level, the unit of randomisation. These estimates will be presented for descriptive purposes only and are not assessments of the validity of randomisation.

Programme impacts will be estimated under the intention-to-treat (ITT) principle using the following cross-sectional regression model for continuous outcomes:

2 $${y}_{ibd}=\alpha +\upbeta {T}_{bd}+{\tau}_{d}+{\varepsilon}_{ibd}$$

where ${y}_{ibd}$ is post-intervention outcome variable (including pre-specified primary and secondary outcomes such as per capita household income, income of persons with disabilities, per capita food consumption and expenditures, and per capita non-food expenditures, etc.); ${T}_{bd}$ is an indicator variable taking the value of 1 if individual or household i is assigned to the treatment group and 0 if assigned to the control group; ${\tau}_{d}$ are district fixed effects and ${\varepsilon}_{ibd}$ are error terms. We will cluster standard error by branch office catchment area level, the unit of randomisation. The key parameter of interest is $\upbeta$ which measures the effect of the programme on the outcome variable.

Employment status is a binary outcome and will be analysed using logistic regression, with treatment assignment included as the primary explanatory variable and district fixed effects to account for stratified randomisation. Standard errors will be clustered at the branch office (catchment) level, the unit of randomisation, using cluster-robust estimators. Results will be reported as odds ratios with corresponding confidence intervals and *p*-values.

The primary model specification is fixed and will not be modified based on statistical significance or model fit.

Given five specified primary outcomes, we will control the family-wise type I error rate at two-sided *α* = 0.05 across the primary outcome family using a Bonferroni correction. Accordingly, each primary outcome hypothesis test will be conducted at a two-sided significance level of *α* = 0.01 (0.05/5). We will report effect estimates and confidence intervals for each primary outcome alongside *p*-values; where confidence intervals are used for inference, Bonferroni-adjusted 99% confidence intervals will be reported alongside effect estimates and *p*-values.

Secondary outcomes will be analysed as exploratory. For these outcomes, we report effect estimates, 95% confidence intervals, and nominal two-sided p-values without formal multiplicity adjustment. Secondary findings will not be used to determine overall intervention effectiveness.

Inference is conducted separately for each outcome within the multiplicity-adjusted framework, and interpretation will emphasise effect magnitude and precision rather than binary declarations of overall “success”.

We will additionally report covariate-adjusted models as supplementary analyses. The equation for models with continuous outcomes is:

$${y}_{ibd}=\alpha +\beta {T}_{bd}+\gamma {X}_{ibd}+{\tau}_{d}+{\varepsilon}_{ibd}.$$

Here, ${X}_{ibd}$ is the baseline value of the post-intervention outcome variable, ${y}_{ibd}$. No additional covariates will be selected. This approach improves statistical precision while preserving the unbiasedness of the treatment effect under random assignment. The same approach will be used for the binary outcome of employment. These adjusted models are interpreted as sensitivity analyses.

Continuous outcomes will be analysed on their natural scale in the primary specification. Where outcomes are substantially right-skewed (e.g. income or expenditure variables), log-transformed models will additionally be reported as robustness analyses. These alternative specifications will be applied consistently across relevant outcomes and reported transparently.

Because assignment may not perfectly determine treatment receipt, the primary model estimates the intention-to-treat (ITT) effect. In the presence of non-compliance, this differs from the effect of actual treatment receipt. As an exploratory analysis, we estimate the local average treatment effect (LATE) using two-stage least squares, with assignment serving as an instrument for treatment receipt.

Due to the 2:1 cluster allocation ratio between the intervention and control arms and variables corresponding to programme exposure, it is not feasible to conduct a blinded analysis of trial data, which represents a limitation of the study.

### Interim analyses {21b}

No interim analyses planned.

### Methods for additional analyses (e.g. subgroup analyses) {20b}

In addition to estimating the average treatment effect, we will also explore heterogeneity in the treatment effects across different subgroups. Specifically, we will test for variations in the programme’s impact based on disability severity and female-headed households and estimate:

$${y}_{ibd}=\alpha +\beta {T}_{bd}+\delta Zibd+\theta (Tbd\times Zibd)+{\tau}_{d}+{\varepsilon}_{ibd}.$$

where ${Z}_{ibd}$ is either baseline disability severity or an indicator for female-headed households. The coefficient $\theta$ tests for differential programme impacts across categories defined by ${Z}_{ibd}$. Standard errors will be clustered at the branch office catchment area level (unit of randomisation), and district fixed effects (${\tau}_{d}$) will be included. Disability severity is defined using scores from the WG SS at baseline. This is a multi-category variable, we will use indicator variables for severity categories (with the lowest severity category as reference). Female-headed households will be defined as households in which the primary decision-maker/household head is female at baseline. These interaction analyses are exploratory.

### Methods in analysis to handle protocol non-adherence and any statistical methods to handle missing data {20c}

The full analysis set (FAS) will be specified according to the intention-to-treat (ITT) principle and will include all randomised participants analysed according to the study arm to which they were assigned at randomisation, irrespective of programme uptake or protocol deviations.. The primary analysis will estimate treatment effects using regression models applied to all available outcome data. Participants with missing outcome data will therefore not contribute to the estimation of treatment effects. This approach assumes that outcomes are missing at random (MAR) conditional on district. We will report the extent of missingness by study arm for each outcome and compare baseline characteristics between participants with observed and missing outcomes to assess potential differential attrition. Where possible, reasons for withdrawal for each group will be reported descriptively by study arm.

### Plans to give access to the full protocol, participant-level data and statistical code {31c}

Twelve months after the end of the study, the anonymised survey data will be made available on LSHTM’s Data Compass (datacompass.lshtm.ac.uk), for which explicit consent has been included in the consent form, alongside project documentation and a data user guide.

## Oversight and monitoring

### Composition of the coordinating centre and trial steering committee {5d}

BRAC is responsible for the intervention design, allocation, implementation, and programme monitoring. BIGD leads data collection for the evaluation, with support from LSHTM. Evaluation design and analysis is led by LSHTM, in partnership with BIGD. Monitoring data collected from BRAC is shared with LSHTM and BIGD for the purpose of the process evaluation.

### Composition of the data monitoring committee, its role and reporting structure {21a}

As the evaluation is not blinded and the interventions pose a limited risk to participants, a data monitoring committee is not required.

### Adverse event reporting and harms {22}

Delivery of the DIUPG Bangladesh programme is monitored by BRAC. The intervention by design is targeted at households who have a member with disability and who meet the criteria for being ultra-poor. This means that many households in villages where the programme is delivered will not receive it as they are ineligible. It is possible that this will generate resentment within the villages. Resentment may lead to perpetration of stigma and discrimination, violence or theft, particularly targeting persons with disabilities. Indeed, infrequent instances of this were observed within the DIUPG Uganda programme [9]. Such events are monitored by BRAC through a feedback and complaint-handling mechanism and will also inform the DIUPG Bangladesh process evaluation.

### Frequency and plans for auditing trial conduct {23}

Data is quality assured in the field by the data collection team supervisor through regular oversight according to protocols, including daily reviews of submitted forms, spot checks during interviews, and direct feedback to data collectors. It is also checked after submission by the data manager. Any discrepancies are followed up with the relevant data collector as required.

### Plans for communicating important protocol amendments to relevant parties (e.g. trial participants, ethical committees) {25}

Any important modifications to the study protocol are agreed between BRAC, BIGD and LSHTM, and approved by LSHTM Ethics Committee and James P Grant School of Public Health Ethics Committee prior to implementation.

### Dissemination plans {31a}

To disseminate findings from this research, we will write academic articles and evidence briefings, present at conferences and publicise the results through other communications within a network of academic and non-academic partners. There will also be community dissemination activities.

## Discussion

The ultra-poor graduation model has been shown to be effective for the mainstream population of the ultra-poor across several different contexts [1] and the UPG approach has been adapted to be disability-inclusive. An evaluation of the DIUPG programme in Uganda showed that the programme successfully increased household consumption expenditures, despite operating amidst challenging conditions such as COVID-19 and funding cuts [9]. There is a general paucity of evidence regarding what interventions work to create better livelihoods for persons with disabilities and their families [6]. Evaluating the DIUPG Bangladesh programme will generate crucial evidence demonstrating whether similar benefits of the disability-inclusive ultra-poor graduation programme in Uganda are observed in the distinct context of Bangladesh. Aside from the empirical gap this addresses, this information is a boon for policymakers looking for successful livelihood approaches that can be adapted and scaled in their setting.

## Trial status

This manuscript is based on protocol version 2, 05/03/2026. Baseline data collection and recruitment were conducted in May–June 2023. The manuscript was updated on 12/09/2025 to reflect that the endline evaluation is planned for September–October 2025, instead of July–August 2025. The manuscript was updated on 05/03/2026 to respond to first-round peer review comments and to clarify that the endline evaluation took place in October–November 2025.

## Supplementary Information

Supplementary Material 1.

## Data Availability

Survey data will be made available on LSHTM’s Data Compass 12 months after the end of the study, along with project documentation and a data-users guide. The data will be made available open access, ensuring that no identifiers are included in the data. Explicit consent has been included for making data open access.

## Abbreviations

BIGD: BRAC Institute of Governance and Development
DIUPG: Disability-inclusive ultra-poor graduation programme
FAS: Full analysis set
ICC: Intraclass correlation coefficient
ITT: Intention-to-treat
LSHTM: London School of Hygiene & Tropical Medicine
MDE: Minimum detectable effect
PENDA: Programme for Evidence to Inform Disability Action
RCT: Randomised controlled trial
SD: Standard deviation
SDG: Sustainable Development Goals
SSNP: Social Safety Net Programmes
ToC: Theory of change
UK: United Kingdom
UPG: Ultra-Poor Graduation programme
VSSC: Village Social Solidarity Committee
WGSS: Washington Group Short Set
